# Association of Workplace Supports With Active Commuting

**Published:** 2010-10-15

**Authors:** Andrew T. Kaczynski, Melissa J. Bopp, Pamela Wittman

**Affiliations:** Department of Kinesiology, Kansas State University; Kansas State University, Manhattan, Kansas; Kansas State University, Manhattan, Kansas

## Abstract

**Introduction:**

Active commuting is associated with a reduced risk of several chronic diseases, but few studies have considered institutional factors that influence it. We examined the association between cultural and physical workplace supports for active commuting and employee active commuting behavior.

**Methods:**

Data were collected from employees (N = 375) in Manhattan, Kansas, via an online survey. Physical and cultural supports for active commuting in the workplace were measured separately. Active commuting frequency was dichotomized as 0 trips versus at least 1 trip per week by foot or bicycle. Binomial logistic regression was used to predict the likelihood of commuting actively at least once per week, according to the number and type of cultural and physical workplace supports. Analyses were conducted by sex and age and for the full sample.

**Results:**

Among the full sample, employees who reported more physical and cultural supports in the workplace for active commuting were more likely to walk or bicycle to work at least once per week. Significant, positive relationships between cultural and physical supports and active commuting were observed for women but not for men. Both younger and older adults who reported 2 or more physical supports in the workplace were more likely to actively commute, but no relationship was observed for cultural supports. The most influential types of individual supports were perceiving that other coworkers actively commute and the presence of bicycle parking and a bicycle storage policy at the workplace.

**Conclusion:**

The presence of workplace physical and cultural supports is related to more active commuting behavior and may especially encourage active commuting among women.

## Introduction

Public health guidelines for physical activity recommend that adults engage in at least 150 minutes of moderate-intensity physical activity per week and that this amount can be accumulated in episodes as short as 10 minutes (1). Rather than relying on recreational activity, which many working people may find difficult to engage in, active commuting, the practice of walking or bicycling to work, offers a promising means to integrate the recommended amount of physical activity into daily routines (2-5).

Much research has linked physical activity with a reduced risk of chronic diseases, including diabetes, cardiovascular disease, and several forms of cancer (1,6). Several studies have documented an association between health and active commuting. Gordon-Larsen et al found a positive association between active commuting and several cardiovascular disease risk factors (7), and similarly, a review of 8 studies demonstrated a robust protective effect of active commuting on cardiovascular outcomes, especially among women (8). A randomized controlled trial of 68 inactive middle-aged men and women revealed that active commuting to work for 1 hour daily for 10 weeks created significant improvements in VO_2max_, maximal treadmill time, and high-density lipoprotein cholesterol (9). Other studies have reported associations between active commuting and obesity (10) and all-cause mortality (11). Aside from benefits to physical health, active commuting has the potential to facilitate positive social (eg, increased contact with other residents), environmental (eg, less pollution), and economic (eg, lower insurance costs) outcomes (12).

Despite these benefits, rates of active commuting in the United States are low; more than 90% of respondents to the 2001 National Household Transportation Survey indicated that an automobile was their usual mode of transportation to work (13). An additional 5% reported taking public transit, almost 3% usually walked, and a little more than 1% used some other form of transportation (including only a portion who regularly bicycled) (13). In contrast, in some European countries, the percentage of trips taken in urban areas by walking or bicycling ranges from 30% to 45% (14). Even Canada, which has arguably poorer weather conditions for nonmotorized travel, exhibits rates of active transportation that are almost double those observed in the United States (14).

Many studies have examined rates and influences on children's active commuting to school (15-17). However, surprisingly little research has examined factors associated with active commuting among adults. One study that used an 18-item self-report measure found that environmental factors (eg, variety of destinations, aesthetics, traffic safety) were positively associated with walking to work, even after controlling for several other variables (18). Another study reported that walking or bicycling to work at least 30 minutes per day was related to participants' perceptions of the utility of active commuting for avoiding parking hassles, reducing expenses, increasing one's health, and reducing air pollution (19). Other research has identified distance as a determinant of active commuting (20,21). However, in general, very little is known about definitive correlates or intervention points for promoting walking and bicycling as an alternative to using cars (22).

Although limited research exists regarding personal and community-level influences on active commuting, few, if any, studies have examined how institutional factors affect an individual's propensity to walk or bicycle to work. Social ecological models posit that intervening at multiple levels is most effective (22), and much research has suggested that institutional or workplace interventions can change health behaviors (23,24). Therefore, we examined the association of cultural and physical workplace supports for active commuting with employee active commuting behavior. Understanding how workplace actions to support active commuting are related to walking and bicycling to work can suggest promising strategies for employer- or employee-initiated worksite interventions to increase utilitarian physical activity and help prevent chronic disease.

## Methods

### Study setting and data collection

This study was conducted in Manhattan, Kansas, a small city (15 sq mi) with a 2006 population of 50,737, approximately three-quarters (76%) of which are adults aged 18 to 64 years (25). In 2006, most of the population was white (87%), 24% of households lived below the federal poverty level, and 95% of adults reported having a high school diploma or higher. The mean travel time to work for people aged 16 years or older was 14 minutes. The major employment sectors were government (41%), service (22%), and retail (20%) (25). At the time of the study, no public transportation system was available in the city.

This study was part of a broader exploratory investigation of active commuting that was approved by the institutional review board at Kansas State University. For the larger study, an online survey was conducted from August to December 2008. Participants were recruited through links from local Web sites (eg, local newspaper, city government bicycle advisory board), e-mails to municipal and school board employees throughout the city, and fliers provided to area employers. Eligibility criteria included being aged 18 years or older, living or working full-time or part-time in Manhattan, Kansas, and being physically able to walk or bicycle. People who began the survey and reported not being employed at all (n = 20) or being physically unable to walk or bicycle (n = 12) were not included in the analyses.

### Measures

The online, anonymous survey (Axio Learning Systems, Manhattan, Kansas) consisted of several pages of questions related primarily to active commuting behaviors. After reading the consent statement, participants were asked during the survey to indicate their sex, age, race, education level, and estimated walking time to work (dichotomized as ≤20 min or >20 min).

Cultural supports for active commuting were measured with 2 Likert scale–type questions. The first asked about respondents' perceptions about the extent to which their employer encourages active commuting, and this was recoded as "absent" ("does not at all encourage" to "a little") versus "present" ("somewhat" to "strongly encourages"). The second question asked about the perceived number of coworkers who actively commute to work (recoded as "none" or "some"). A dichotomous variable was created that categorized participants as having neither type of cultural support or having at least 1 cultural support.

Physical supports for active commuting were measured with 3 yes/no questions about the availability of bicycle parking, bicycle storage policies, and showers or lockers at the workplace. These variables were summed to designate respondents with 0, 1, or 2 or more of the 3 types of physical supports. Participants were also asked to indicate the number of times per week they walked and bicycled to or from work, and a dichotomous active commuting outcome variable was created indicating 0 trips versus at least 1 trip by foot or bicycle. All survey questions relevant to this study are provided in the Appendix.

### Analysis

Chi-square tests were used to assess differences in the prevalence of active commuting behavior and physical and cultural supports between men and women and between respondents of different ages (18-39 y vs ≥40 y). Binomial logistic regression was used to predict the likelihood of actively commuting to work at least once per week, according to the number of cultural and physical workplace supports reported, using the 0 supports category as the reference group for each model. We also examined the likelihood of active commuting on the basis of the presence or absence of the 5 types of supports. For all analyses, separate models were examined for the full sample of participants, for men and women independently, and for younger (18-39 y) and older (≥40 y) adults independently. All analyses controlled for age, race, education, perceived walking time to work, and sex (when these variables were not used to stratify the sample). Missing data were few but excluded pairwise. All analyses were conducted using SPSS version 17.0 (SPSS, Inc, Chicago, Illinois).

## Results

In total, 375 people completed the survey (Table 1). The mean age of respondents was 40 years, and most participants were white, female, and highly educated, which was largely representative of the study city (25). Approximately one-quarter of the sample reported actively commuting to or from work at least once per week, and men and younger adults were more likely to do so (Table 2). Approximately three-quarters of the sample reported that their workplace possessed at least 1 cultural support for active commuting. Men were more likely than women to report the presence of at least 1 cultural support. Approximately one-third of the full sample reported their workplace had no physical supports for active commuting. Again, men were more likely than women to report the presence of at least 2 physical supports, and there were few substantial differences in perceptions of physical supports by age group (Table 2).

In the full sample, participants who reported 2 or more physical supports for active commuting were more likely to actively commute at least once per week than those who reported none, but participants reporting only 1 physical support were not (Table 3). When the sample was stratified by sex, disparate results were observed. For men, neither having 2 or more nor a single physical workplace support was related to increased odds of active commuting. In contrast, compared with women who reported no physical supports in the workplace, women who reported 2 or more physical supports were more than 10 times as likely to actively commute, and women who reported a single physical support were more than 3 times as likely to actively commute. When the sample was stratified by age, compared with participants who reported no physical supports, both younger (18-39 y) and older (≥40 y) participants were more than 3 times as likely to actively commute if 2 or more physical supports were present in the workplace. Reporting only 1 physical support was not associated with increased odds of active commuting for either age group (Table 3).

In the full sample, participants who reported 1 or more cultural supports were more than twice as likely to actively commute at least once per week as participants who reported no cultural supports (Table 3). Women who reported having 1 or more cultural supports in the workplace were more than twice as likely to walk or bicycle to work at least once per week as women who reported having no cultural supports. However, this was not true of men. No significant differences were found between participants who reported 1 or more cultural supports in the workplace and participants who reported no cultural supports when the sample was stratified by age (Table 3).

We also examined the combined and disaggregated effects of different types of supports. Among the full sample, participants who reported at least 1 cultural support and at least 1 physical support for active commuting in the workplace were more than 6 times as likely to actively commute at least once per week as participants who reported both types of supports absent (Table 3). This cumulative effect was consistent for women but not for men or either age group of adults.

For all 5 samples examined, perceiving that the employer encourages active commuting was not related to increased odds of walking or bicycling to work at least once per week, and having showers or lockers at the workplace was related to increased odds of active commuting only for women (Table 4). However, the other 3 individual supports — perceptions of the number of coworkers who actively commute, availability of bicycle parking, and having bicycle storage policies — were generally related to an increased likelihood of active commuting, although bicycle parking was not a significant factor for men or older adults.

## Discussion

We found that people with a workplace environment that had more cultural and physical supports were more likely to walk or bicycle to work. However, this relationship between workplace supports and active commuting behavior appeared to be moderated by sex, as such associations were significant only for women in our sample. For women, the presence of at least 1 cultural support, in particular their perceptions that other coworkers walk or bicycle to work, was associated with almost a threefold increase in the likelihood of active commuting at least once per week. Likewise, women who reported 1 physical support were more than 3 times as likely to actively commute, while women with 2 or 3 physical supports were 10 times as likely to do so. Furthermore, the cumulative effect of having at least 1 cultural and at least 1 physical workplace support was strongest for women in this study. To our knowledge, few, if any, studies have explored how influences on active commuting differ by sex. One study (26) reported that women had stronger preferences for community infrastructure for bicycling (eg, off-road routes with separation from motorized traffic), and the same may be true for workplace supports for active commuting. Additionally, women may be more concerned with cleanliness and tidiness of their appearance compared with men, explaining the importance of the availability of showers and lockers to support active commuting. Women generally report lower levels of overall physical activity and lower self-efficacy for physical activity (27), but additional qualitative and experimental research is needed to further explore the reasons why workplace supports appear to differentially benefit active commuting among women.

When the sample was stratified by age, the presence of 2 or more physical workplace supports was associated with a greater likelihood of active commuting for both younger and older adults. However, with respect to cultural supports, the significant relationship observed for the full sample failed to materialize for either the younger or older subgroups. When considered in isolation, though, greater perceptions of the number of coworkers who actively commute was related to increased active commuting behavior for both age groups. Having a larger percentage of fellow employees who walk or bicycle to work may create an environment that tolerates, fosters, or reinforces similar behavior among colleagues. Future research can shed light on the psychological (eg, modeling, affiliation) or behavioral (eg, "walkpooling" or "bicyclepooling") mechanisms behind the relevance of having coworkers who actively commute.

The primary strength of our study was its novelty in considering supports in workplace settings that may influence active commuting. However, our study does have limitations. First, our data were collected from a convenience sample that was small. Because the exact number of people who were eligible and had the opportunity to complete the survey is unknown, we were unable to calculate a response rate. We also could not entirely rule out the possibility that respondents were clustered in workplaces, although some data collected in the survey (closest intersection to workplace) suggested that the participants originated from various locations. Future studies may examine our research questions with a larger, random sample of employees, potentially in a multilevel framework. Second, we used self-report measures of both the workplace environment and active commuting behavior (although perceived measures of workplace supports may be more important than objective indicators). Third, our online survey format may have excluded potential respondents who did not have access to the Internet. Finally, our sample was well-educated and almost exclusively white, which limits the generalizability of our findings.

Our results substantiate the impact of a social-ecological approach for promoting active commuting. Many studies have examined the utility of workplace interventions for promoting physical activity, with mixed results. For example, Proper et al (28) reviewed 26 studies of worksite interventions to promote physical activity or fitness and found evidence of a positive effect of such programs on physical activity and musculoskeletal disorders and found limited to inconclusive evidence for a positive effect on fatigue, physical fitness, general health, blood serum lipids, and blood pressure. Dishman et al (29) also reviewed 26 studies that delivered physical activity interventions through worksites and concluded that the mean effect of the interventions was heterogeneous and small. However, most studies included in those reviews employed largely individual-level approaches (eg, goal setting) to promote leisure-time (rather than utilitarian) physical activity. Our findings indicate that providing a supportive physical and cultural environment that promotes active commuting is associated with higher rates of walking and bicycling to work, which in turn can have significant health benefits (7-11).

In terms of health promotion, our results provide a foundation for intervention strategies, including potential physical changes to workplaces, such as the addition of showers, bicycle racks, and covered and secure bicycle parking. Likewise, a workplace culture that supports active commuting can influence mode of travel to work. The social support that results when a large number of workers actively commute, either collectively or individually, can be reinforcing (30). Such practices may be facilitated by team challenges or other worksite events focused around active commuting. Employer and government policies may also foster a climate supportive of active commuting. For example, offering tax breaks, parking refunds, health insurance premium reductions, or other financial incentives may encourage active commuting among employees. In combination with traditional individually targeted approaches, these institutional strategies can make active commuting more attractive. Moreover, our data suggest that such strategies may be especially influential for encouraging active commuting among women, a subgroup that generally exhibits lower rates of active transportation and overall physical activity (31). As workplaces increasingly adopt such practices, documenting the costs of such investments in comparison with the savings enjoyed by both employees (eg, reduced health care costs) and employers (eg, reduced sick time) is important.

Our study adds to the small body of existing literature concerning factors associated with active commuting among adults and contributes to our understanding of social-ecological influences on this behavior beyond the individual. More research is needed to evaluate interventions that aim to promote active commuting (20), but making changes to workplace infrastructure and policies may be effective avenues for increasing rates of active commuting and improving employee health.

## Figures and Tables

**Table 1:** Sample Characteristics, 375 Employees in Manhattan, Kansas, Fall 2008

Characteristic	No. (%)
**Sex**	
Male	147 (39)
Female	227 (61)
Missing data	1 (0)
**Age, y**	
18-39	191 (51)
≥40	179 (48)
Missing data	5 (1)
**Education**	
High school or less	20 (5)
More than high school	353 (94)
Missing data	2 (0)
**Race**	
White	335 (89)
Nonwhite	36 (10)
Missing data	4 (1)
**Walk time to work, min**	
≤20	86 (23)
>20	279 (74)
Missing data	10 (3)

**Table 2:** Differences in Reported Active Commuting Frequency, Cultural Supports, and Physical Supports, by Sex and Age, 375 Employees in Manhattan, Kansas, Fall 2008

Sample Group	Actively Commute to or From Work at Least Once Per Week				No. of Cultural Supports^a^				No. of Physical Supports^b^				

	Yes, %	No, %	χ^2^	*P* Value	0, %	≥1, %	χ^2^	*P* Value	0, %	≥1, %	≥2, %	χ^2^	*P* Value
Full sample	26	74	—	—	24	76	—	—	30	35	36	—	—
Men	37	63	11.23	<.001	16	84	6.71	.01	24	33	43	6.07	.04
Women	20	80			28	72			33	36	31		
Aged 18-39 y	32	68	5.48	.02	24	76	0.21	.64	35	32	33	1.50	.47
Aged ≥40 y	21	79			22	78			34	28	38		

a Out of a possible 2 cultural supports (employer encourages active commuting, perception that there are coworkers who actively commute).

b Out of a possible 3 physical supports (bicycle parking available, bicycle storage policies in place, showers/lockers present).

**Table 3:** Relationship Between Physical and Cultural Workplace Supports and Likelihood of Active Commuting, by Sex and Age, 375 Employees in Manhattan, Kansas, Fall 2008^a^

Reported No. of Physical and Cultural Workplace Supports	Full Sample, β (95% CI)	Men, β (95% CI)	Women, β (95% CI)	Aged 18-39 y, β (95% CI)	Aged ≥40 y, β (95% CI)
No physical supports^b^	1 [Reference]	1 [Reference]	1 [Reference]	1 [Reference]	1 [Reference]
1 Physical support^b^	0.97 (0.43-2.20)	0.29 (0.08-1.00)	3.74 (1.08-8.87)	0.73 (0.23-2.34)	0.77 (0.20-2.97)
≥2 Physical supports^b^	3.62 (1.71-7.69)	1.88 (0.67-5.28)	10.30 (2.74-18.73)	3.45 (1.23-9.71)	3.79 (1.17-12.29)
No cultural supports^c^	1 [Reference]	1 [Reference]	1 [Reference]	1 [Reference]	1 [Reference]
≥1 Cultural supports^c^	2.56 (1.19-5.99)	2.17 (0.69-8.87)	2.83 (1.23-6.21)	2.38 (0.77-7.34)	2.41 (0.65-8.99)
Both supports absent	1 [Reference]	1 [Reference]	1 [Reference]	1 [Reference]	1 [Reference]
At least 1 physical and at least 1 cultural support	6.42 (1.38-19.80)	1.12 (0.17-7.25)	5.39 (1.58-14.25)	2.49 (0.71-8.13)	2.31 (0.45-11.72)

Abbreviation: CI, confidence interval.

a Outcome variable dichotomized as 0 trips versus at least 1 trip to or from work per week by foot or bicycle.

b Out of a possible 3 physical supports (bicycle parking available, bicycle storage policies in place, showers/lockers present).

c Out of a possible 2 cultural supports (employer encourages active commuting, perception that there are coworkers who actively commute).

**Table 4:** Relationship Between Individual Cultural and Physical Workplace Supports and Likelihood of Active Commuting, by Sex and Age, 375 Employees in Manhattan, Kansas, Fall 2008^a^

Type of Individual Support	Full Sample ,β (95% CI)	Men, β (95% CI)	Women, β (95% CI)	Aged 18-39 y, β (95% CI)	Aged ≥40 y, β (95% CI)
Employer encourages active commuting	0.62 (0.31-1.20)	0.83 (0.31-2.23)	0.44 (0.16-1.17)	0.70 (0.26-1.89)	0.53 (0.19-1.48)
Perception that there are coworkers who actively commute	3.98 (1.91-8.28)	4.04 (1.32-12.39)	4.23 (1.56-11.51)	3.73 (1.36-10.30)	3.97 (1.36-11.56)
Bicycle parking available	2.70 (1.40-5.21)	1.72 (0.70-4.26)	5.54 (1.87-11.39)	2.97 (1.22-7.22)	2.23 (0.81-6.13)
Bicycle storage policies in place	5.92 (3.03-11.58)	5.04 (1.97-12.93)	8.35 (3.10-22.53)	6.68 (2.58-17.29)	5.19 (2.00-13.41)
Showers/lockers present	0.86 (0.42-1.72)	0.49 (0.18-1.31)	2.72 (1.12-4.46)	0.43 (0.13-1.40)	1.50 (0.60-3.73)

Abbreviation: CI, confidence interval.

a Outcome variable dichotomized as 0 trips versus at least 1 trip to work per week by foot or bicycle.

## Appendix

### Online Survey Questions Related to Active Commuting, 375 Employees in Manhattan, Kansas, Fall 2008

**Table:**

How many of your coworkers actively commute (walk or bicycle) to your workplace?	None/A few/Some/Most
Does your employer encourage active commuting?	1 = Does not at all encourage to 5 = Strongly encourages
Does your employer offer bicycle parking?	Yes/No/Don't know
Are there policies for bicycle storage at your workplace?	Yes/No/Don't know
Are there locker rooms or shower facilities at your workplace?	Yes/No/Don't know
Thinking about the past month: On average, how many days per week do you drive to work in an automobile driven by yourself or with someone else?	0-7 d/wk
Thinking about the past month: On average, how many days per week do you drive from work in an automobile driven by yourself or someone else?	0-7 d/wk
Thinking about the past month: On average, how many days per week do you walk to work?	0-7 d/wk
Thinking about the past month: On average, how many days per week do you walk from work?	0-7 d/wk
Thinking about the past month: On average, how many days per week do you bicycle to work?	0-7 d/wk
Thinking about the past month: On average, how many days per week do you bicycle from work?	0-7 d/wk
